# Associations of *MMP9* polymorphism with the risk of severe pneumonia in a Southern Chinese children population

**DOI:** 10.1186/s12879-023-08931-4

**Published:** 2024-01-02

**Authors:** Li Cai, Xiaoyu Zuo, Liuheyi Ma, Yuxia Zhang, Falin Xu, Bingtai Lu

**Affiliations:** 1 Department of Hospital Infection Control, The Second Affiliated Hospital of Guangzhou University of Chinese Medicine, Guangzhou, Guangdong 510120 China; 2 Department of Pediatric Surgery, Guangzhou Institute of Pediatrics, Guangzhou Women and Children’s Medical Center, Guangzhou Medical University, Guangzhou, Guangdong 510623 China; 3 Department of Pediatrics, The Third Affiliated Hospital of Zhengzhou University, Zhengzhou, Henan Province 450052 China; 4 Medical Research Institute, Guangdong Provincial People’s Hospital (Guangdong Academy of Medical Sciences), Southern Medical University Guangzhou, Guangzhou, Guangdong 510080 China

**Keywords:** Pneumonia, *MMP9*, Single nucleotide polymorphism

## Abstract

**Background:**

Severe pneumonia frequently causes irreversible sequelae and represents a major health burden for children under the age of 5. Matrix Metallopeptidase 9 (MMP9) is a zinc-dependent endopeptidase that is involved in various cellular processes. The correlation between MMP9 and the risk of severe childhood pneumonia remains unclear.

**Methods:**

Here we assemble a case–control cohort to study the association of genetic variants in *MMP9* gene with severe childhood pneumonia susceptibility in a Southern Chinese population (1034 cases and 8426 controls).

**Results:**

Our results indicate that the allele G in rs3918262 SNP was significantly associated with an increased risk of severe pneumonia. Bioinformatic analyses by expression quantitative trait loci (eQTL), RegulomeDB and FORGEdb database analysis showed that rs3918262 SNP has potential regulatory effect on translational efficiency and protein level of *MMP9* gene. Furthermore, MMP9 concentrations were significantly up-regulated in the bronchoalveolar lavages (BALs) of children with severe pneumonia.

**Conclusion:**

In summary, our findings suggest that *MMP9* is a novel predisposing gene for childhood pneumonia.

**Supplementary Information:**

The online version contains supplementary material available at 10.1186/s12879-023-08931-4.

## Background

Community-acquired pneumonia (CAP) is an infectious lung disease and particularly affects children under 5 years of age [1–3]. Severe CAP is also a leading cause of childhood morbidity and mortality worldwide, with an estimated 120 million cases per year, of which about 1.3 million cases cause mortality [4]. Children with severe pneumonia often develop various sequelae, including chronic obstructive pulmonary diseases (COPD), bronchiolitis obliterans (BO), pulmonary fibrosis, bronchiectasis, and asthma [5–7]. The cause of childhood pneumonia is complicated. Viral and bacterial infections were the primary environmental cause of severe pneumonia [8].

The matrix metallopeptidase 9 (MMP9) is a member of the matrix metalloproteinase (MMP) enzyme family, which is defined by the zinc ion in the catalytic domain [9]. The MMP enzyme family plays an essential role in the degradation and regulation of extracellular matrix (ECM) proteins [10]. MMP9 belongs to the gelatinase subtype of MMP [11], and is involved in multiple biological processes including proteolytic ECM degradation [12], cell migration and invasion [13], cell adhesion [14], cell interaction [15], apoptosis [16], and angiogenesis [17]. Notably, circulating MMP9 level has been reported as a biomarker for severe CAP [18], COVID-19 [19], ventilator-associated pneumonia [20] and acute respiratory distress syndrome (ARDS) [21]. Mechanistically, MMP9 may contribute to inflammation by promoting the infiltration of leukocyte and proinflammatory macrophages, as demonstrated in experimental glomerulonephritis [22]. A recent study showed that the skin lesion infiltrating neutrophils in psoriasis overexpressed the *MMP9* gene and secreted MMP9 protein. MMP9 activates vascular endothelial cells through MAPK signaling pathways and enhances CD4^+^T cell transmigration in psoriatic pathogenesis [23].

It has been shown that polymorphism in *MMP9* were associated with breast cancer risk [24] and chronic venous disease [25]. In addition, *MMP9* polymorphism has been suggested to play protective roles in diabetic microvascular complications and pressure ulcers [26, 27]. In summary, MMP9 is associated with inflammatory diseases and is a biomarker for severe pneumonia. However, the associations between *MMP9* polymorphism and severe childhood pneumonia need further elucidation.

In order to further investigate the association between *MMP9* polymorphism and CAP susceptibility, we conducted a case-control study including a South Chinese population with 1034 cases and 8624 controls to verify the effects of selected SNP. Our results suggest epistatic association of *MMP9* rs3918262 SNPs and severe childhood pneumonia.

## Methods

### Study subjects

We recruited 1034 cases and 8624 controls from Guangzhou Women and Children’s Medical Center. Remnant venous blood samples after clinical examination were collected from the patients. The study was approved by the Medical Ethics Committees of the recruiting hospitals (BF2022-257–01, 2016111853, KY-Q-2021–165-02). Information on demographic characteristics, disease severity, and pathogens were retrieved from each patient’s electronic medical record (EMR).

### Inclusion and exclusion criteria of the study subjects

The inclusion criteria for cases were as follows: (1) Age < 16 years old; (2) Patients who were diagnosed with severe pneumonia or non-severe pneumonia according to a published criteria [28]: (a) invasive mechanical ventilation; (b) fluid refractory shock; (c) acute need for noninvasive positive-pressure ventilation; and (d) hypoxemia requiring a fraction of inspired oxygen (FiO_2_) > inspired concentration or flow feasible in the general-care area.

The exclusion criteria were as follows: (1) known or suspected active tuberculosis; (2) primary immunodeficiency; (3) acquired immunodeficiency syndrome (AIDS) and immunosuppressive medications taken before admission; (4) lack of eligible data or blood samples. Control subjects were recruited from the physical examination centers without a medical history of pneumonia, respiratory diseases, immunodeficiency, and autoimmune disease.

Severe pneumonia was further categorized into two subtypes: Diagnosis as primary pneumonia was defined as primary pneumonia caused by pathogens such as viruses, bacteria, fungus, or mycoplasma. Diagnosis as secondary severe pneumonia was defined as secondary pneumonia caused by other diseases such as cardiovascular diseases or injury.

### DNA extraction and genotyping

Genomic DNA from the venous blood samples were extract by TIANamp Blood DNA Kits (Tiangen Biothch, DP335-02, Beijing, China) kit according to the manufacturer’s instructions. The purity and concentrations of DNA were examined by a NanoPhotometer ® N50 (Implen GmbH., Munich, Germany). Then extracted DNA was amplified by an ABI-7900 real-time quantitative PCR instrument (Applied Biosystems, Foster City, CA, USA).

### SNP based association analysis

Common SNPs within ± 5 kb flanking of *MMP9* were retrieved from “dbSnp153Common” database by using UCSC hgTable, and filtered by the minor allele frequency (MAF > 0.05) in the East Asian (EAS) population. The pairwise linkage disequilibrium (LD) between SNPs were accessed in 1000 Genome EAS population and visualized as LD heatmap by using R package “LDheatmap” [29]. A *R*^2^ > 0.8 was viewed as high LD. Tag-SNPs were selected as the minimal set of independent SNPs that represent all SNPs in each LD block.

### SNP genotyping and quality control

A Common *MMP9* variants were selected using the GTEx portal website (http://www.gtexportal.org/home/) to predict potential associations between the SNP and *MMP9* expression levels. For the 1034 severe childhood pneumonia cases and 8624 controls, SNPs were genotyped using a MassARRAY iPLEX Gold system (Sequenom). Hardy–Weinberg equilibrium tests were performed.

### Statistical analysis

The *χ*^2^ test was applied to test the SNP genotypes for Hardy–Weinberg equilibrium in the control population. The allelic association test examined the difference in allelic frequency distribution between cases and controls. Univariate and multivariate logistic regression models were used to examine the association between genotypes and phenotypes under multiple genetic models, such as additive, codominant, and dominant models. Age and sex were adjusted in the multivariate logistic regression. Odds ratios (ORs) and 95% confidence intervals (CIs) were calculated as the effect sizes. *P* < 0.05 was deemed statistically significant. All analyses were performed by using PLINK (v1.9b).

### Bioinformatic analysis

Furthermore, several in silico analyses were conducted to investigate the functional implication of the *MMP9* polymorphism. GTEx portal was used to assess the expression quantitative trait locus (eQTL) effects of the SNP (https://gtexportal.org/home/). The selection was made according to the RegulomeDB and FORGEdb database. We kept SNPs with high probability to be regulatory variants (RegulomeDB score higher than 2; https://regulomedb.org/ and FORGEdb score higher than 5; https://forge2.altiusinstitute.org/files/forgedb.html).

### MMP9 levels measured by ELISA

Criteria for pneumonia severity and the collection of bronchoalveolar lavage samples were described in our previous study [30]. Bronchoalveolar lavage samples were collected from patients who diagnosed as primary pneumonia and grouped as severe and non-severe patients according to the criteria above. BAL samples were centrifuged at 12,000 rpm for 5 min, and the supernatant was stored at -40 °C until subsequent measurement in ELISA. The expression level of MMP9 was detected according to the manufacturer’s instructions. Briefly, 100 μl of BAL supernatant was added to an antigen-coated plate and incubated with biotinylated detection antibodies and HRP-conjugated molecules. After a 20-min incubation with substrate reagents, the absorbance was measured at 450 nm using an enzyme-linked immunosorbent assay reader (Thermo), and the grouping criteria were the same as before. Non-parametric Kruskal–Wallis test was utilized to compare the differences among the control group, mild pneumonia group, and severe pneumonia group, and *p*-value < 0.05 was considered statistically significant.

## Results

### Study subjects

In this study, we assembled a cohort including 1034 severe childhood pneumonia cases and 8426 healthy controls. The demographic features of the participants are shown in Table 1. Patients were aged between 2 days to 15 years, of whom 61.2% were male. Viral (69.7%), bacterial (60.4%) and fungus (10.4%) infections were detected among most of the patients. The percentages of mixed viral-bacterial, viral-fungus, bacterial-fungus, and viral-bacterial-fungus co-infections were 41.1%, 7.4%, 8.0%, and 5.4%, respectively. 75.0% of the patients had received mechanical ventilation, and 94.6% had comorbid conditions. 719 (69.5%) patients had the primary diagnosis as severe pneumonia while others (315, 30.5%) were secondary pneumonia.

**Table 1:** Demographics of patients

	**Severe pneumonia (*n* = 1034)**	**Controls (*n* = 8426)**
Age, median (range), years	0.6 (2 days–15 years)	6 (1 month–20 years)
Gender, n (%)		
Male	660 (62.1)	4735 (56.2)
Female	374 (37.9)	3691 (43.8)
Identifier pathogens, n (%)		
Viral	721 (69.7)	N/A
Bacterial	625 (60.4)	N/A
Fugus	108 (10.4)	N/A
Viral + Bacteria	425 (41.1)	N/A
Viral + Fugus	77 (7.4)	N/A
Bacteria + fugus	83 (8.0)	N/A
Viral + Bacteria + Fugus	56 (5.4)	N/A
Comorbid conditions, n (%)	978 (94.6)	N/A
Mechanical ventilation	776 (75.0)	N/A
Diagnosis as primary severe pneumonia	719 (69.5)	N/A
Diagnosis as secondary severe pneumonia	315 (30.5)	N/A

*N/A* Unavailable data

### Screening of potential regulatory SNPs in *MMP9*

**Fig. 1:**
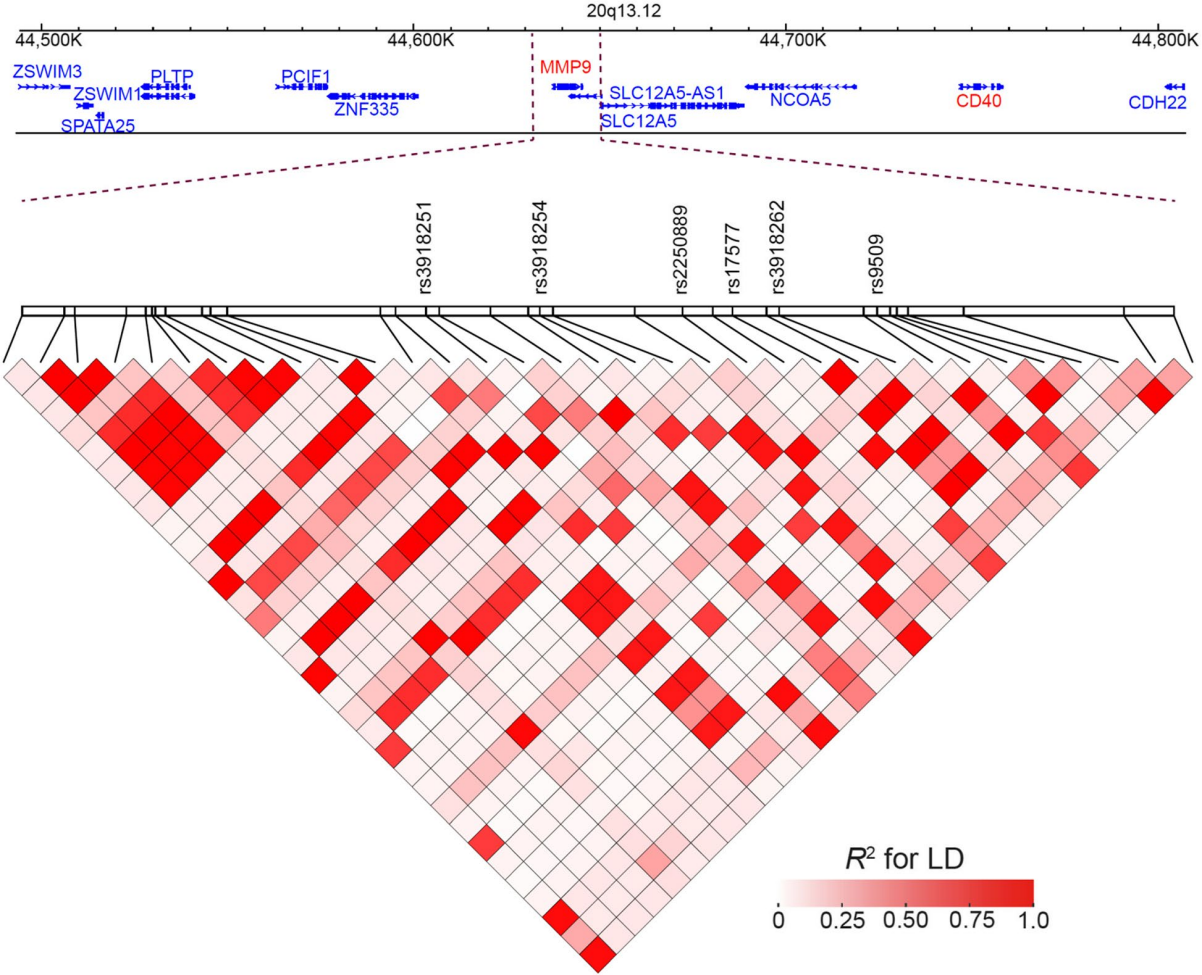
Linkage disequilibrium (LD) heatmap for SNPs with high regulatory potential in *MMP9*.* R*^2^ was used to quantify the scale of LD

We first screened the common SNPs within 5 kb upstream and downstream region of *MMP9* gene via UCSC platform. 16 candidate SNPs were further analyzed using linkage disequilibrium (LD) patterns with *R*^2^ > 0.8 as a cut-off, and 6 tagSNPs (rs3918251, rs3918254, rs2250889, rs17577, rs3918262, rs9509) in *MMP9* gene were selected for genotyping (Fig. 1 and Table S1).

We then examined the possible regulatory functions of *MMP9* SNPs using RegulomeDB and FORGEdb database. RegulomeDB scores of 1–6 indicate most to least likely to affect binding and expression of target gene were assigned for each of the SNPs. We identified 2 SNPs in MMP9, rs3918262 and rs17577, which showed evidence of strong regulatory potential with a score of 2b (Table 2). The other 4 SNPs in MMP9, rs3918251, rs2250889, rs3918254 and rs9509, exhibited less likely regulatory potential (RegulomeDB score = 4–6). Next we used FORGEdb database to predict the possibility of the *MMP9* SNPs to be a regulatory variant. FORGEdb scores of from 0 to 10 and higher scores indicate greater likelihood that the SNP is a regulatory variant. Consistent with RegulomeDB results, rs3918262 and rs17577 had a high FORGEdb score of 9 and 10. Thus, we selected rs3918262 and rs17577 as prioritized for further evaluation in this study.

**Table 2:** Regulation potentials of risk alleles of *MMP9* gene SNP

**RS_Number**	**Position**	**Alleles**	**MAF**	**Distance**	**Dprime**	**R2**	**Correlated_Alleles**	**FORGEdb**^**a**^	**RegulomeDB**^**b**^	**Function**
**rs3918262**	chr20:44643770	(A/G)	0.370	4989	1.000	0.332	A = A, G = G	8	2b	NA
**rs17577**	chr20:44643111	(G/A)	0.168	4330	0.166	0.010	NA	10	2b	missense
rs3918251	chr20:44638781	(A/G)	0.361	0	1.000	1.000	A = A, G = G	8	4	NA
rs2250889	chr20:44642406	(G/C)	0.250	3625	1.000	0.591	G = A, C = G	8	4	missense
rs3918254	chr20:44640391	(C/T)	0.188	1610	0.929	0.112	C = A, T = G	9	4	NA
rs9509	chr20:44645153	(T/C)	0.221	6372	0.478	0.037	NA	8	6	NA

5 SNPs with high regulatory potentials were selected by securing FORGEdb and RegulomeDB. SNPs selected for subsequent analyses were marked in bold

*Abbreviations*: *MAF* Minor Allele Frequency, MAF was presented as (MAF in cases)/(MAF in controls), *Dprime* SNP haplotype frequency, *R2* SNP correlation, Correlated alleles refer to alleles that are correlated if linkage disequilibrium is present (*R*2 > 0.1). rs3918251 was used as reference SNP

^a^FORGEdb scores indicated the regulatory potentials for SNPs (https://forge2.altiusinstitute.org/files/forgedb.html). FORGEdb scores of from 0 to 10 and higher scores indicate greater likelihood that the SNP is a regulatory variant

^b^RegulomeDB scores indicated the regulatory potentials for SNPs (https://regulomedb.org/regulome-search). RegulomeDB scores of 1–6 indicate most to least likely to affect binding and expression of target gene. A score 2b denotes that the SNP was within a potential regulatory region supported by TF binding, binding motif, DNase Foorprint, and DNase peak data

### Associations between *MMP9* polymorphism and the risk of severe pneumonia

Compared with the control subjects, we found that rs3918251, rs2250889 and rs3918262 showed statistical significance after Bonferroni correction for multiple testing. The minor A allele frequency of rs3918251 (0.26 vs. 0.30, *OR* = 0.83, 95% CI = 0.75–0.92, *P* = 0.0006), G allele frequency of rs2250889 (0.19 vs. 0.22, *OR* = 0.84, 95% CI = 0.74–0.94, *P* = 0.0023) and A allele frequency of rs2250889 (0.11 vs. 0.13, *OR* = 0.84, 95% CI = 0.73–0.97, *P* = 0.0198) was significantly associated with decreased risk of severe pneumonia. In contrast, the minor G allele frequency of rs3918262 (0.45 vs. 0.42, *OR* = 1.16, 95% CI = 1.05–1.27, *P* = 0.0024) and rs3918254 (0.22 vs. 0.20, *OR* = 1.13, 95% CI = 1.01–1.27, *P* = 0.0279) were significantly associated with increased risk of severe pneumonia (Table 3). Moreover, rs3918251 and rs3918262 SNPs have prominent effects on homozygous children compared with that on heterozygous children, whereas rs17577 affects heterozygous individuals (*p* < 0.0042) (Table 3).

**Table 3:** Risk allele frequency of *MMP9* gene SNPs in the case-control data set of children with severe pneumonia

**CHR**	**BP**	**SNP**	**Major allele**	**Minor allele**	**Minor Allele Frequency in Cases**	**Minor Allele Frequency in Controls**	**Test**	**OR (95% CI)**	***P***
**20**	44638781	rs3918251	G	A	0.265	0.302	Allelic	0.832 (0.748–0.925)	0.0006
							Heterozygous	0.867 (0.755–0.996)	0.0442
							Homozygous	0.651 (0.496–0.854)	0.0019
**20**	44642406	rs2250889	C	G	0.191	0.220	Allelic	0.843 (0.744–0.938)	0.0023
							Heterozygous	0.841 (0.730–0.969)	0.0165
							Homozygous	0.698 (0.496–0.983)	0.0392
**20**	44643770	rs3918262	A	G	0.453	0.418	Allelic	1.156 (1.052–1.269)	0.0024
							Heterozygous	1.019 (0.877–1.184)	0.8066
							Homozygous	1.374 (1.143–1.652)	0.0007
**20**	44643111	rs17577	G	A	0.114	0.132	Allelic	0.835 (0.731–0.973)	0.0198
							Heterozygous	0.782 (0.664–0.921)	0.0032
							Homozygous	1.082 (0.666–1.759)	0.7496
**20**	44640391	rs3918254	C	T	0.218	0.198	Allelic	1.135 (1.014–1.271)	0.0279
							Heterozygous	1.068 (0.927–1.232)	0.3619
							Homozygous	1.474 (1.091–1.991)	0.0114
**20**	44645153	rs9509	T	C	0.232	0.219	Allelic	1.082 (0.970–1.207)	0.1588
							Heterozygous	1.016 (0.883–1.168)	0.8280
							Homozygous	1.326 (1.001–1.756)	0.0491

*P* value adjusted by gender and age. The *P*-values were corrected by Bonferroni’s method with a threshold of 0.0042. Calculation of the OR was also based on the minor allele of each SNP

*Abbreviations*: *CHR* Chromosome, *BP* Base pair (where the SNP is located), *SNP* Single-nucleotide polymorphism, *OR* Odds ratio, *CI* Confidence interval

### Nominal significance of *MMP9* SNPs with severe pneumonia subtype

To explore the association between *MMP9* polymorphism and the severe pneumonia subtype, we further divided our cohort into two subgroups: the primary diagnosis as severe pneumonia (*n* = 719) and the secondary diagnosis as severe pneumonia (*n* = 315). Results showed that the minor G allele of rs3918262 SNP showed evidence of a trend towards significance (*P* = 0.08) between those subgroups (Table 4). None of the other 5 SNPs (rs3918251, rs3918254, rs2250889, rs17577, rs9509) of *MMP9* gene were significantly different between two group (Table 4).

**Table 4:** Risk allele frequency of *MMP9* gene SNPs between severe pneumonia subgroups

**CHR**	**BP**	**SNP**	**Major allele**	**Minor allele**	**Minor Allele Frequency in primary pneumonia**	**Minor Allele Frequency in secondary pneumonia**	**OR (95% CI)**	***P***
**20**	44643770	rs3918262	A	G	0.440	0.484	0.846 (0.699–1.026)	0.0888
**20**	44643111	rs17577	G	A	0.120	0.010	1.212 (0.889–1.654)	0.2231
**20**	44638781	rs3918251	G	A	0.270	0.250	1.118 (0.888–1.409)	0.3420
**20**	44642406	rs2250889	C	G	0.196	0.181	1.110 (0.865–1.426)	0.4114
**20**	44640391	rs3918254	C	T	0.222	0.209	1.074 (0.846–1.363)	0.5565
**20**	44645153	rs9509	T	C	0.233	0.229	1.020 (0.812–1.282)	0.8628

The *P*-values were corrected by Bonferroni’s method with a threshold of 0.0042. Calculation of the OR was also based on the minor allele of each SNP

*Abbreviations*: *CHR* Chromosome, *BP* Base pair (where the SNP is located), *SNP* Single-nucleotide polymorphism, *OR* Odds ratio, *CI* Confidence interval, *P P*-value adjusted by gender and age

### Expression quantitative trait loci (eQTLs) of *MMP9* SNPs

To identify functional effects of *MMP9* variants, we conducted expression quantitative trait loci (eQTL) analysis on 6 SNPs of *MMP9*. As shown in Table 5, eQTL analysis indicated that the minor alleles of rs3918251, rs17577 and rs3918262 were not only associated with the increased mRNA expression (Effect size > 0) of MMP9, and but also its vicinity gene CD40 (Fig. 1). From GTEx database, those SNPs affected *MMP9* and CD40 mRNA expression within subcutaneous adipose, skin and testis, whereas the minor allele of rs3918251 may downregulate the mRNA expression of *MMP9* in cultured fibroblasts (Table 5).

**Table 5:** eQTL results curated from GTEx database for SNPs on of risk alleles of MMP9 gene

**RS ID**	**R2**	**Gene Symbol**	**Tissue**	**Effect Size**	***P***
rs3918251	1	*MMP9*	Cells - Cultured fibroblasts	-0.203	8.55E-08
rs3918251	1	CD40	Adipose - Subcutaneous	0.156	1.51E-05
rs3918251	1	*MMP9*	Skin - Not Sun Exposed (Suprapubic)	0.259	2.46E-07
rs3918251	1	*MMP9*	Skin - Sun Exposed (Lower leg)	0.182	4.12E-05
rs3918251	1	CD40	Skin - Sun Exposed (Lower leg)	0.136	1.20E-04
rs17577	1	*MMP9*	Testis	0.348	8.02E-05
rs3918261	1	*MMP9*	Testis	0.340	1.16E-04
rs3918262	1	CD40	Adipose - Subcutaneous	0.181	2.65E-05
rs3918262	1	CD40	Skin - Sun Exposed (Lower leg)	0.177	4.05E-05

Functional relevance of SNP on gene expression in GTEx database. Significant eQTL results of MMP9 SNPs on the nearby genes were summarized

*eQTL* Expression quantitative trait loci, *Effect size* The degree to which an SNP influences the expression of genes in a particular tissue

### Levels of MMP9 in the bronchoalveolar lavages (BALs) of children with severe pneumonia

**Fig. 2:**
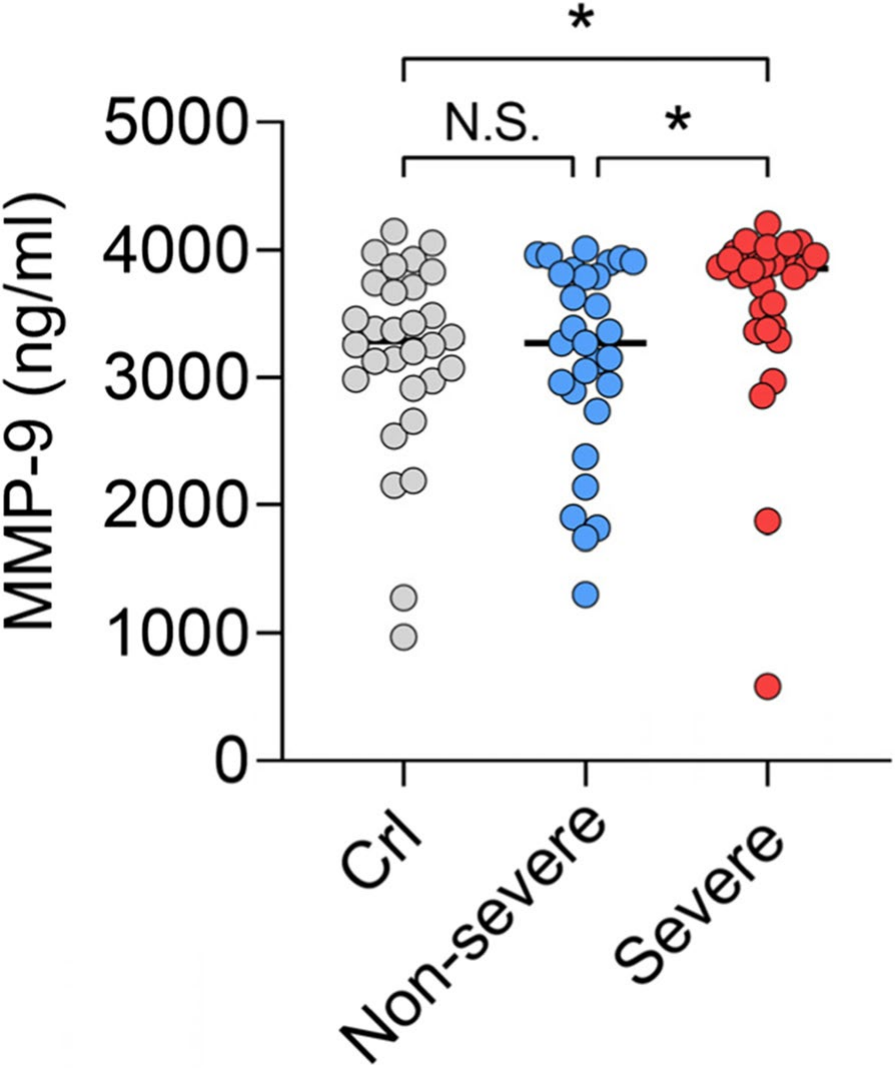
MMP9 concentrations in the BALs of children in control group (*n* = 30), non-severe pneumonia group (*n* = 30) and severe pneumonia group (*n* = 29). (*:*P* < 0.05)

In order to validate the levels of MMP9 proteins in the lung of children with severe pneumonia, we measured MMP9 concentrations by ELISA within the BAL samples freshly collected from children with primary pneumonia, with control samples taken from patients without a concurrent infection but requiring surgical removal of inhaled foreign objects. Patients were further grouped as severe and non-severe patients according to the criteria in Methods. We found that compared with control and non-severe subjects, the MMP9 levels were significantly increased in severe pneumonia patients, whereas no significant difference was found between the control and non-severe groups (Fig. 2). We further evaluated the effects of the MMP9 SNPs on the measured MMP9 protein levels from ELISA in the severe patient group. BAL MMP9 levels were significantly increased in patients with rs3918251 AG/AA genotype when compared with GG genotype (*P* < 0.05), whereas the remaining SNPs show no significant association with the expression of MMP9 protein (*P* value ranged from 0.124 to 0.815) (sFig. 1).

## Discussion

Community-acquired pneumonia is a common cause of morbidity and mortality in children under 5 years [31]. Although most of the cases are self-limiting disease, around 13% of severe pneumonia cases in PICU unfortunately pass away [32]. Serious sequelae, such as bronchiolitis obliterans (BO), pulmonary fibrosis and bronchiectasis [5–7], are another burden for those who recover from severe pneumonia. Risk factors of severe pneumonia are numerous, including age, immunodeficiencies, malnutrition, chronic lung diseases, and cystic congenital thoracic malformations [33]. Previous research has documented the existence of gender disparities in community-acquired pneumonia [34]. Consistent with these findings, our cohort also exhibited a gender distinction, as over 50% of the patients were male.

The matrix metalloproteinase (MMP) family plays essential roles in lung organogenesis and the pathogenesis of inflammatory diseases [35]. Dysregulation of MMP contributes to a series of lung tissue damage and disorders such as asthma [36], idiopathic pulmonary fibrosis (IPF) [37], emphysema [38], ARDS [39] and COPD [40]. MMP are also involved in the remodeling of lung after inflammation [41, 42]. Among MMP family members, matrix metalloproteinase MMP9 is enriched in lungs of asthma, IPF and COPD, and also promotes lung remodeling [43]. Associations are also found for *MMP9* polymorphisms with lung cancer [44], COPD [45] and asthma [46].

In this study, we first screened the common SNP within *MMP9* gene and located 6 candidate SNPs (Table S1). Then we identified rs3918262 and rs17577 as priority SNP by RegulomeDB and FORGEdb analysis (Table 2). Notably, our study showed that the G allele frequency of rs3918262 was significantly associated with increased risk of severe pneumonia (Table 3). No previous study has mentioned the function of this allele. Moreover, rs3918262 showed evidence of a trend towards significance between patients primary or secondary diagnosed as severe or pneumonia (Table 4). Secondary pneumonia was defined as pneumonia caused by non-respiratory reasons [47]. This finding suggests that the minor allele of G of rs3918262 is potentially associated with infections.

From expression quantitative trait loci (eQTL) analysis, we found SNP rs17477 and rs3918262 of *MMP9* gene potential regulatory effect on *MMP9* or CD40 expression. As CD40 gene is located downstream of the *MMP9* gene and in its vicinity, these SNPs may influence the expression of the CD40 gene. In addition, CD40 has been reported to be associated with inflammatory immune diseases such as Kawasaki disease [48], suggesting its potential relevance in regulating pneumonia as well. Most of the alleles exhibit up-regulated effects as the effect sizes were > 0. However, no significant difference was detected in lung tissue. Nevertheless, results from other organs suggest that these SNPs may up-regulate the protein expression of MMP9 or CD40.

Additionally, eQTL, RegulomeDB and FORGEdb results suggest that rs17577, which is located in the coding region of *MMP9* gene, had strong regulatory potential on gene expression. Rs17577 was reported to be associated with pediatric asthma [49, 50], breast cancer [51], and ischemic stroke [52] etc. Although the *P*-value (*P* = 0.0198) of rs17577 SNP between control and pneumonia group did not meet the threshold (0.00083) of Bonferroni’s correction, this SNP may have important impact on *MMP9* expression and potentially was a risk factor of severe pneumonia. It should be noted that no previous study reported about the regulatory functions of the minor allele of G of rs3918262.

Finally, we measure the MMP9 protein levels in the BAL of children with severe or non-severe pneumonia. Results showed that the MMP9 concentrations were up-regulated in severe patients (Fig. 2). In the literature, MMP9 is produced by activated alveolar macrophages and neutrophils, catalyzes the proteolysis of the extracellular matrix and plays a role in leukocyte migration [53, 54]. Taken together with our regulatory results, MMP9 SNPs may contribute to severe pneumonia susceptibility through regulatory properties which may impact *MMP9* translational efficiency and protein level. However, these remain to be investigated experimentally in the future. Finally, we demonstrated that protein levels of MMP9 were significantly increased in the BAL of patient with severe pneumonia, suggesting that *MMP9* polymorphism may associated with the expression levels of MMP9 in severe pneumonia.

There are some limitations for this study. First, only children were included in our cohort which may reduce the statistical power of the study. Second, the regulatory effects of the SNPs should be validated by subsequent experiments. Finally, only Chinese Han subjects from Southern China were enrolled in this study, and the conclusions should be extrapolated to other ethnic groups with caution.

## Conclusions

Nevertheless, this study indicates that the polymorphisms in *MMP9* are associated with the susceptibility to severe pneumonia in Southern Chinese children. Future study may elucidate mechanisms of action and reveal whether *MMP9* may serve as a potential biomarker or therapeutic target for severe childhood pneumonia.

### Supplementary Information

**Additional file 1: Table S1.** Final SNP Clip.

**Additional file 2: Supplemental figure 1.** BAL MMP9 levels of severe pneumonia patients with rs3918251 GG of AG/AA genotype (*:*P* < 0.05).

## Data Availability

The datasets generated during and analysed during the current study are available from the corresponding author on reasonable request.

## Abbreviations

AIDS: Acquired immunodeficiency syndrome
ARDS: Acute respiratory distress syndrome
BALs: Bronchoalveolar lavages
BO: Bronchiolitis obliterans
BP: Base pair
CAP: Community-acquired pneumonia
CD: Cluster of differentiation
CHR: Chromosome
CIs: Confidence intervals
COPD: Chronic obstructive pulmonary
COVID-19: Coronavirus disease 2019
DNA: Deoxyribonucleic acid
EAS: East Asian
ECM: Extracellular matrix
ELISA: Enzyme linked immunosorbent assay
EMR: Electronic medical record
eQTL: Expression quantitative trait loci
FiO_2_: Fraction of inspired oxygen
HRP: Horseradish peroxidase
HWE: Hardy-Weinberg equilibrium
IPF: Idiopathic pulmonary fibrosis
LD: Linkage disequilibrium
MAF: Minor Allele Frequency
MAPK: Mitogen-activated protein kinase
MMP9: Matrix Metallopeptidase 9
MMP: Matrix metalloproteinase
mRNA: Messenger RNA
OR: Odds ratios
PCR: Polymerase chain reaction
PICU: Pediatric intensive care unit
RNA: Ribonucleic acid
REF: Major allele
SARS-CoV-2: Severe acute respiratory syndrome coronavirus 2
SNP: Single nucleotide polymorphism
