# Supplementary material for: Associations of MMP9 polymorphism with the risk of severe pneumonia in a Southern Chinese children population
Source: BMC Infect Dis. 2024 Jan 2;24:19. doi: 10.1186/s12879-023-08931-4 (PMC10763005; doi:10.1186/s12879-023-08931-4)
Supplement: Supplementary file 1 — Additional file 1: Table S1. Final SNP Clip. [file 12879_2023_8931_MOESM1_ESM.docx]

**Table S1 Final SNP Clip**

| RS Number | Position | Alleles | Details |  |
| --- | --- | --- | --- | --- |
| rs9509 | chr20:44645153 | T=0.779, C=0.221 | Variant kept. |  |
| rs17577 | chr20:44643111 | G=0.832, A=0.168 | Variant kept. |  |
| rs3918262 | chr20:44643770 | A=0.63, G=0.37 | Variant kept. |  |
| rs2250889 | chr20:44642406 | G=0.25, C=0.75 | Variant kept. |  |
| rs3918254 | chr20:44640391 | C=0.812, T=0.188 | Variant kept. |  |
| rs3918251 | chr20:44638781 | A=0.361, G=0.639 | Variant kept. |  |
| rs13969 | chr20:44642833 | A=0.772, C=0.228 | Variant in LD with rs9509 (R^2^=0.9638), variant removed. | |
| rs3787268 | chr20:44641731 | G=0.635, A=0.365 | Variant in LD with rs3918262 (R^2^=0.9795), variant removed. | |
| rs3918250 | chr20:44638352 | A=0.811, G=0.189 | Variant in LD with rs3918254 (R^2^=0.9896), variant removed. | |
| rs17576 | chr20:44640225 | A=0.288, G=0.712 | Variant in LD with rs2250889 (R^2^=0.8222), variant removed. | |
| rs3918249 | chr20:44638136 | T=0.288, C=0.712 | Variant in LD with rs2250889 (R^2^=0.8222), variant removed. | |
| rs3918261 | chr20:44643592 | A=0.832, G=0.168 | Variant in LD with rs17577 (R^2^=1.0), variant removed. | |
| rs13925 | chr20:44644965 | G=0.832, A=0.168 | Variant in LD with rs17577 (R^2^=1.0), variant removed. | |
| rs2236416 | chr20:44640575 | A=0.843, G=0.157 | Variant in LD with rs17577 (R^2^=0.9209), variant removed. | |
| rs2274755 | chr20:44639692 | G=0.843, T=0.157 | Variant in LD with rs17577 (R^2^=0.9209), variant removed. | |
