# Supplementary figures and images for: Associations of MMP9 polymorphism with the risk of severe pneumonia in a Southern Chinese children population
Source: BMC Infect Dis. 2024 Jan 2;24:19. doi: 10.1186/s12879-023-08931-4 (PMC10763005; doi:10.1186/s12879-023-08931-4)

**Supplemental figure 1. BAL MMP9 levels of severe pneumonia patients with rs3918251 GG of AG/AA genotype. (*:*P*<0.05)**


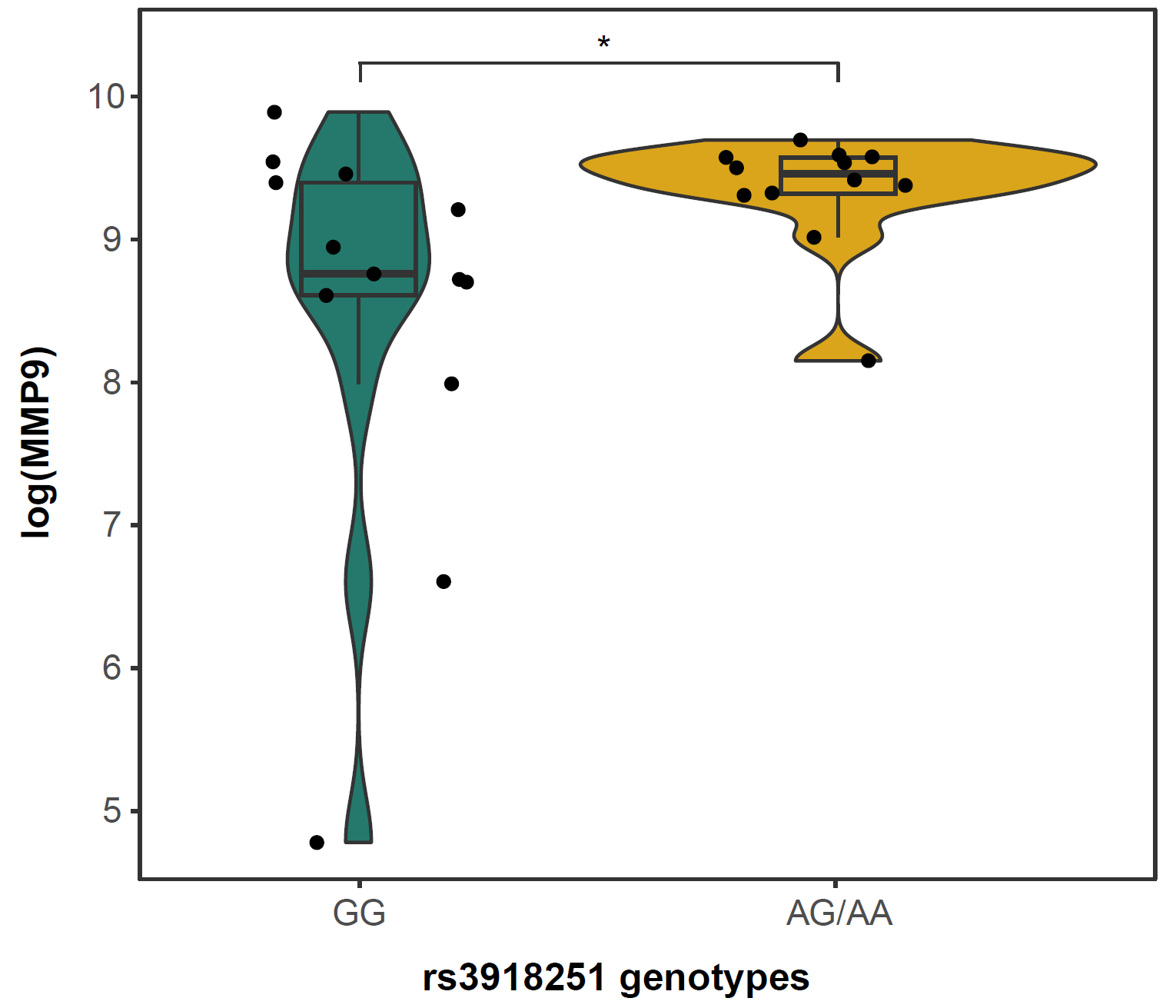

Supplement: Supplementary file 2 — Additional file 2: Supplemental figure 1. BAL MMP9 levels of severe pneumonia patients with rs3918251 GG of AG/AA genotype (*:P < 0.05). [file 12879_2023_8931_MOESM2_ESM.docx]
